# Supplementary material for: Interstitial lung disease diagnosis and prognosis using an AI system integrating longitudinal data
Source: Nat Commun. 2023 Apr 20;14:2272. doi: 10.1038/s41467-023-37720-5 (PMC10119160; doi:10.1038/s41467-023-37720-5)
Supplement: Supplementary file 1 — Supplementary Information [file 41467_2023_37720_MOESM1_ESM.docx]

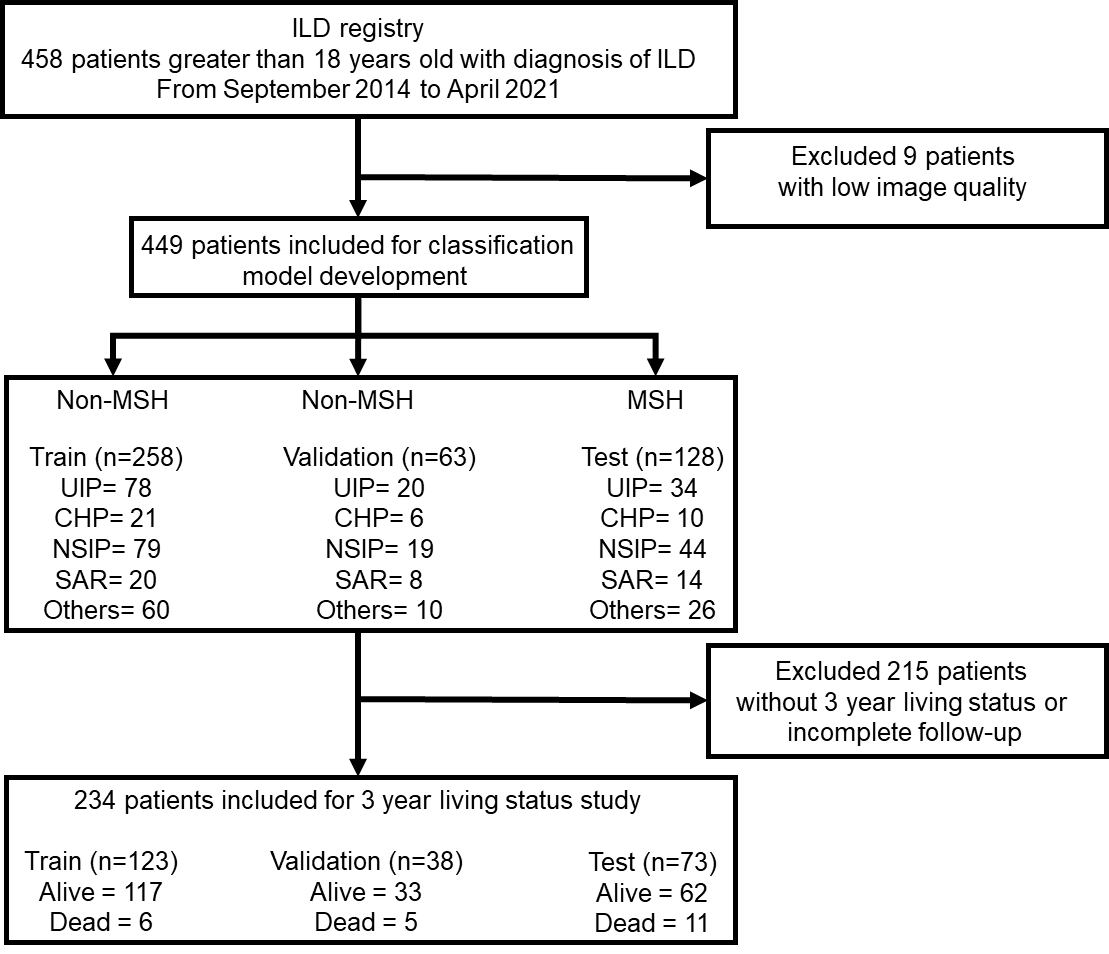


**Supplementary Fig. 1** | Patient inclusion and exclusion criteria.


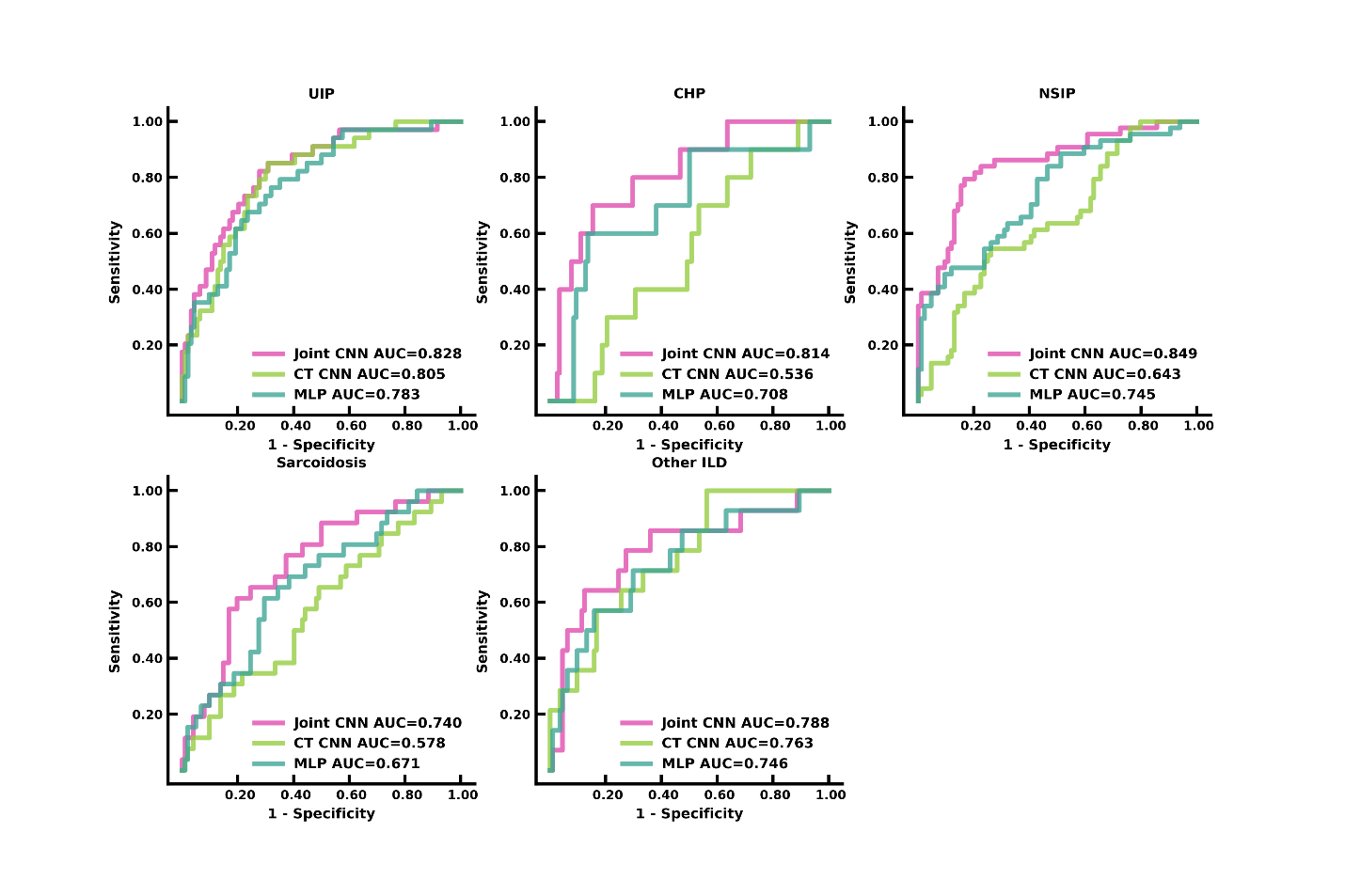


**Supplementary Fig. 2** | The results of joint CNN, CT-only CNN, and MLP models on the ILD classification. AUC comparison of these three models on each ILD subtype in the test set of 128 cases.


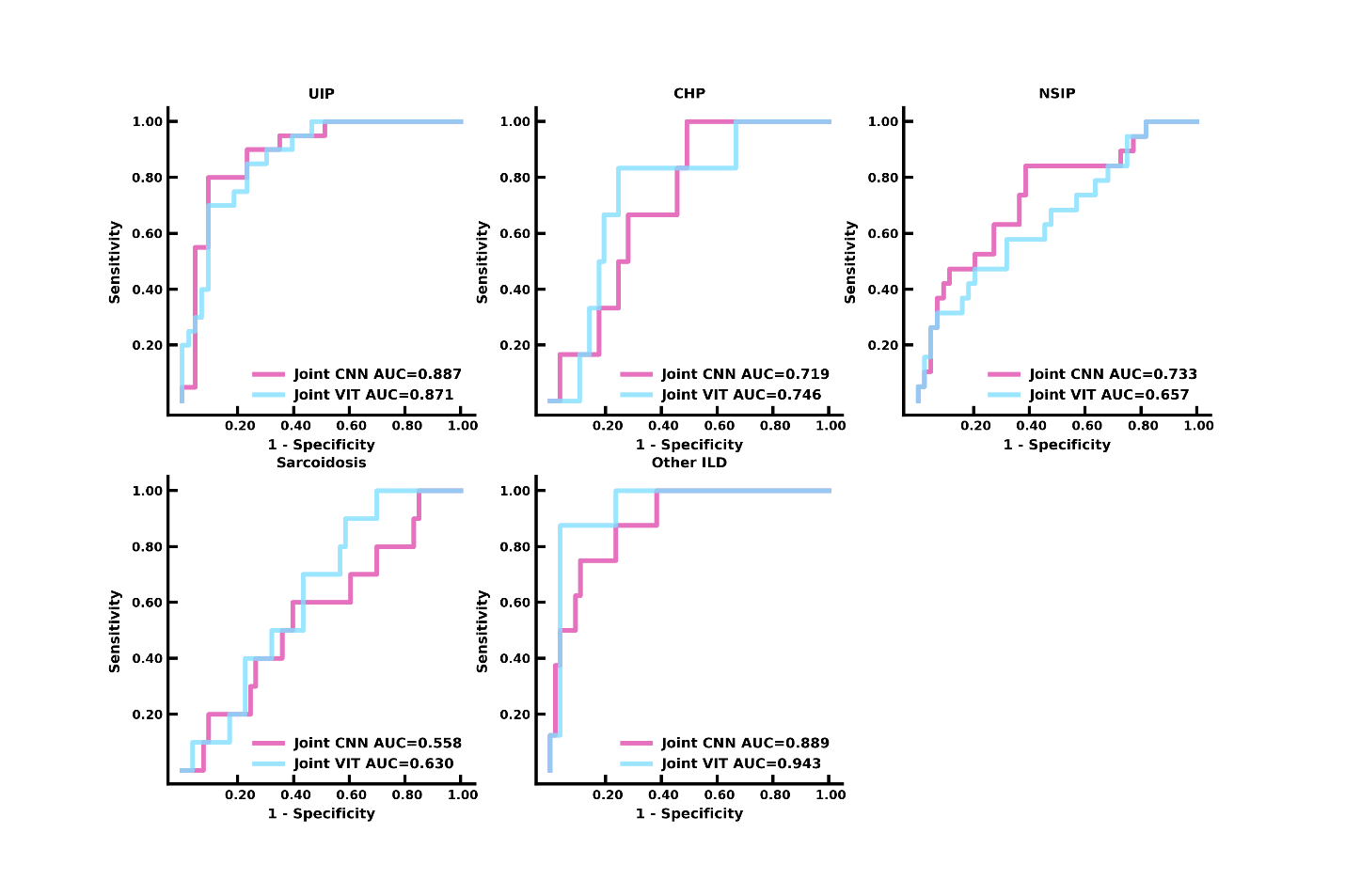


**Supplementary Fig. 3** | The results of joint CNN and joint ViT models on the ILD classification. AUC comparison of two models on each ILD subtype in the validation set of 63 cases.


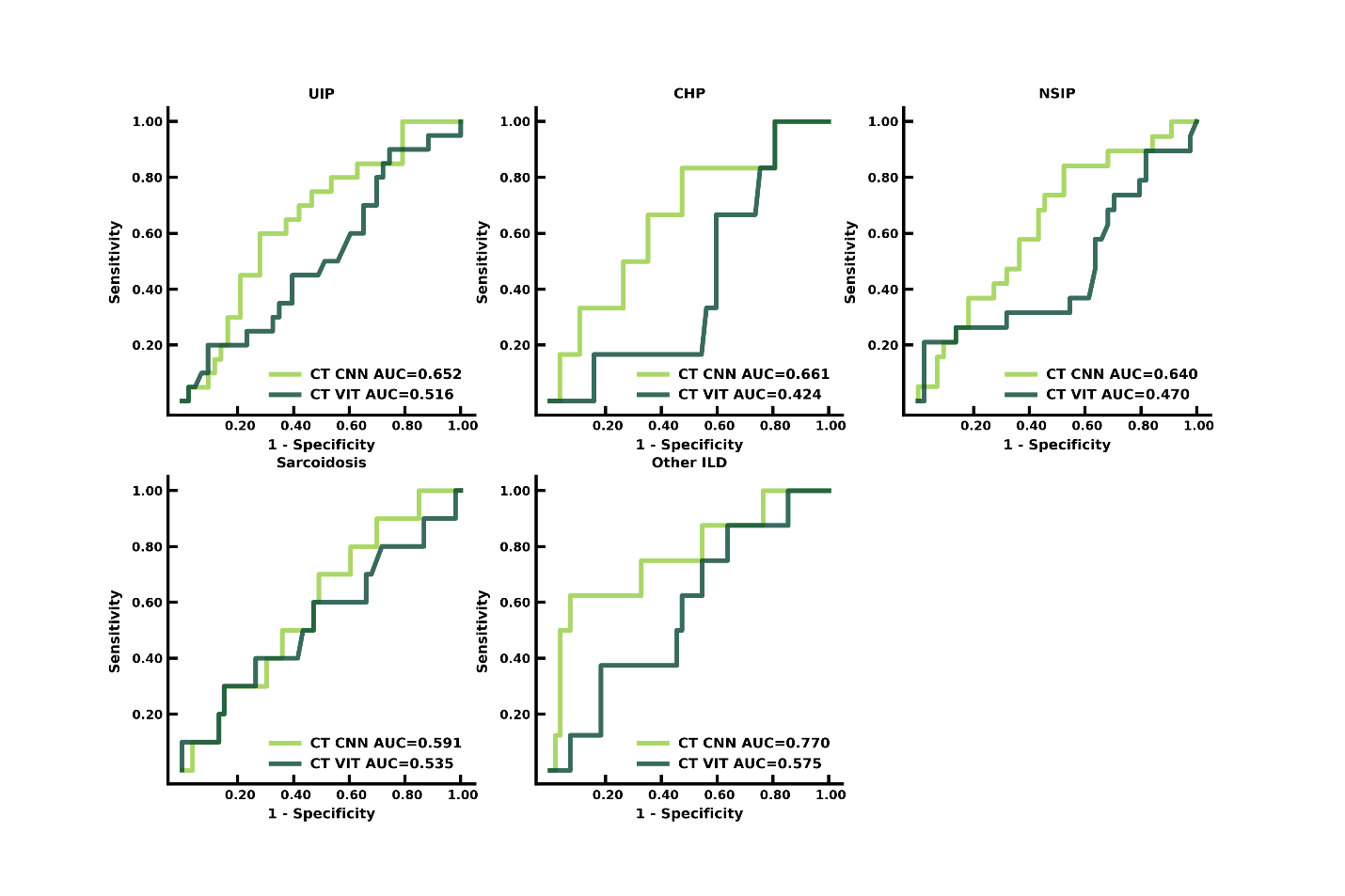


**Supplementary Fig. 4** | The results of CT-only CNN and CT-only ViT models on the ILD classification. AUC comparison of two models on each ILD subtype in the validation set of 63 cases.


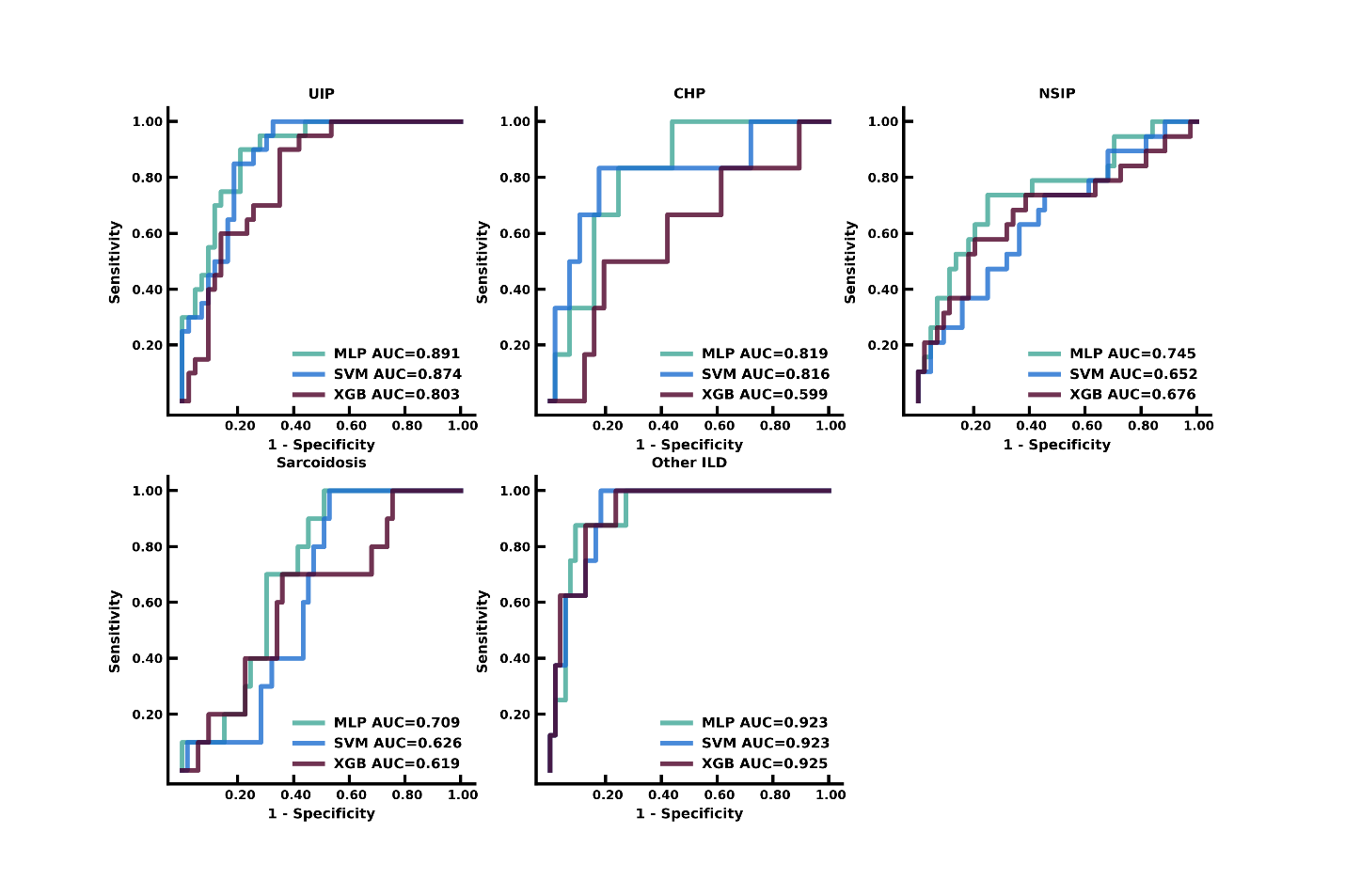


**Supplementary Fig. 5** | The results of MLP, SVM, and XGBoost models on the ILD classification. AUC comparison of these three models on each ILD subtype in the validation set of 63 cases.


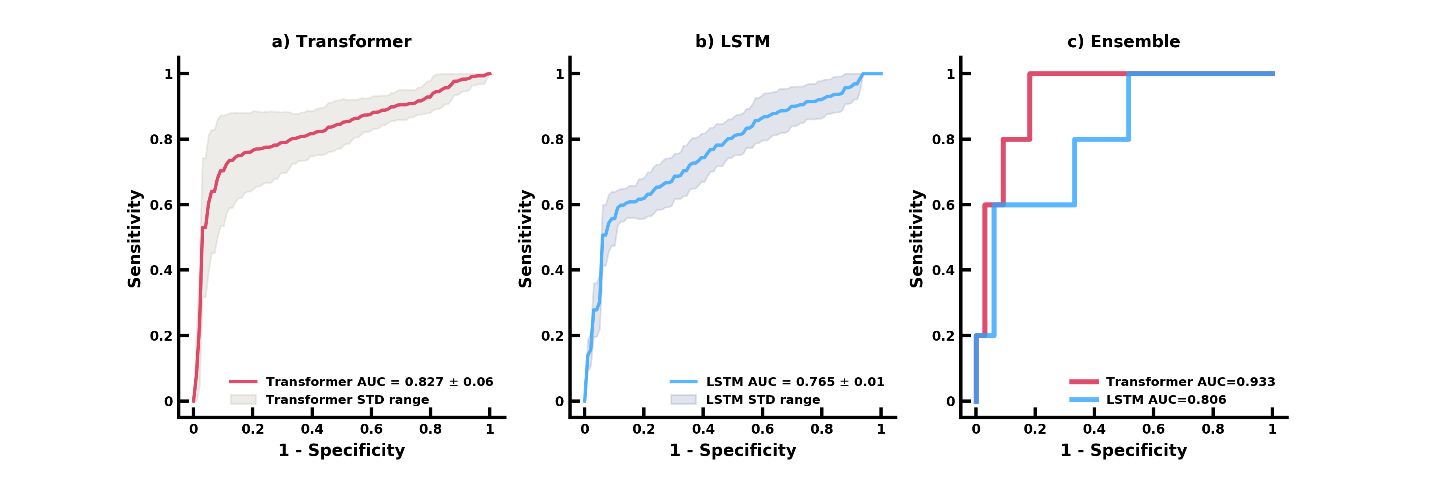


**Supplementary Fig. 6** | LSTM vs Transformer at Year 3 time point in the validation set of 38 cases. a,b) The ROC curves for the average of 60 simulations of LSTM and Transformer models developed on two parameter settings. The shades indicate standard deviation obtained by averaging false positive rates and true positive rates from each model. c) The ROC curves for the ensemble model of LSTM and Transformer models. In figure a) and b), data are presented as mean AUC +/- SD.

**Supplementary Table. 1** | Comparisons of AUC, positive and negative predictive values between the joint AI model and human readers. Data in parenthesis are 95% CI.

1. UIP

|  | AUC | Positive Predictive Value | Negative Predictive Value |
| --- | --- | --- | --- |
| Senior Thoracic radiologist | 0.763  (67.69-85.00) | 86.36  (66.66-95.25) | 85.85  (80.58-89.87) |
| Junior thoracic radiologist1 | 0.685  (59.82-77.10) | 77.78  (55.31-90.82) | 81.82  (77.20-87.60) |
| Junior thoracic radiologist1 | 0.703  (61.47-79.21) | 72.73  (53.21-86.21) | 83.02  (78.00-87.08) |
| Thoracic radiology fellow | 0.810  (72.76-89.31) | 75.00  (59.90-85.77) | 89.58  (83.58-93.56) |
| Senior radiologist1 | 0.674  (58.06-76.80) | 51.43  (38.29-64.37) | 82.80  (76.89-87.44) |
| Senior radiologist2 | 0.626  (54.54-70.62) | 71.43  (45.64-88.16) | 78.95  (75.04-82.39) |
| Senior pulmonologist | 0.679  (59.23-76.63) | 73.68  (52.17-87.79) | 81.65  (76.99-85.55) |
| Joint AI model | 0.828  (74.77-90.88) | 48.28  (40.07-56.68) | 91.43  (83.59-95.72) |

1. CHP

|  | AUC | Positive Predictive Value | Negative Predictive Value |
| --- | --- | --- | --- |
| Senior Thoracic radiologist | 0.682  (51.6-84.85) | 23.81  (12.65-40.28) | 95.33  (91.62-97.44) |
| Junior thoracic radiologist1 | 0.569  (41.61-72.28) | 13.64  (5.32-30.72) | 93.40  (90.34-95.53) |
| Junior thoracic radiologist1 | 0.524  (38.91-65.84) | 10.00  (2.91-29.18) | 92.59  (90.08-94.51) |
| Thoracic radiology fellow | 0.649  (48.68-81.15) | 25.00  (11.63-45.78) | 94.64  (91.39-96.71) |
| Senior radiologist1 | 0.512  (41.1-61.28) | 10.00  (1.54-44.16) | 92.37  (90.73-93.74) |
| Senior radiologist2 | 0.482  (37.94-58.5) | 5.88  (0.91-29.77) | 91.89  (90.11-93.38) |
| Senior pulmonologist | 0.624  (46.04-78.7) | 18.18  (8.52-34.66) | 94.34  (90.90-96.53) |
| Joint AI model | 0.814  (67.57-95.14) | 40.00  (11.16-77.96) | 93.50  (91.33-95.15) |

1. NSIP

|  | AUC | Positive Predictive Value | Negative Predictive Value |
| --- | --- | --- | --- |
| Senior Thoracic radiologist | 0.701  (61.79-78.47) | 66.67  (52.60-78.28) | 78.26  (72.02-83.43) |
| Junior thoracic radiologist1 | 0.631  (54.33-71.86) | 52.38  (40.42-64.08) | 74.42  (67.90-80.00) |
| Junior thoracic radiologist1 | 0.620  (53.2-70.72) | 51.22  (39.10-63.20) | 73.56  (67.19-79.08) |
| Thoracic radiology fellow | 0.678  (59.33-76.28) | 62.16  (48.53-74.11) | 76.92  (70.69-82.17) |
| Senior radiologist1 | 0.612  (53.84-68.56) | 68.42  (46.93-84.15) | 71.56  (67.3-75.46) |
| Senior radiologist2 | 0.657  (56.96-74.43) | 50.00  (41.02-58.98) | 78.57  (70.27-85.05) |
| Senior pulmonologist | 0.683  (60.3-76.39) | 57.14  (45.88-67.89) | 84.21  (74.32-90.77) |
| Joint AI model | 0.849  (77.66-92.2) | 78.38  (64.46-87.87) | 83.52  (76.96-88.49) |

1. Sarcoidosis

|  | AUC | Positive Predictive Value | Negative Predictive Value |
| --- | --- | --- | --- |
| Senior Thoracic radiologist | 0.674  (54.37-80.47) | 83.33  (38.59-97.55) | 92.62  (89.47-94.89) |
| Junior thoracic radiologist1 | 0.701  (56.58-83.64) | 66.67  (35.97-87.69) | 93.28  (89.80-95.63) |
| Junior thoracic radiologist1 | 0.706  (57.05-84.06) | 75.00  (40.07-93.08) | 93.33  (89.89-95.66) |
| Thoracic radiology fellow | 0.701  (56.58-83.64) | 66.67  (35.97-87.69) | 93.28  (89.80-95.63) |
| Senior radiologist1 | 0.670  (53.34-80.74) | 37.50  (20.47-58.31) | 92.86  (89.17-95.36) |
| Senior radiologist2 | 0.674  (54.37-80.47) | 83.33  (38.59-97.55) | 92.62  (89.47-94.89) |
| Senior pulmonologist | 0.679  (54.83-80.88) | 100.00  (100.00-100.00) | 92.68  (89.55-94.93) |
| Joint AI model | 0.788  (64.25-93.27) | 50.00  (27.17-72.83) | 93.10  (89.54-95.51) |

1. Other ILD

|  | AUC | Positive Predictive Value | Negative Predictive Value |
| --- | --- | --- | --- |
| Senior Thoracic radiologist | 0.627  (51.99-73.42) | 32.56  (23.17-43.59) | 85.88  (79.78-90.36) |
| Junior thoracic radiologist1 | 0.560  (45.53-66.46) | 27.03  (17.12-39.91) | 82.42  (77.20-86.65) |
| Junior thoracic radiologist1 | 0.584  (47.83-68.98) | 29.73  (19.49-42.51) | 83.52  (78.15-87.77) |
| Thoracic radiology fellow | 0.599  (49.37-70.39) | 32.35  (21.20-45.95) | 84.04  (78.85-88.15) |
| Senior radiologist1 | 0.554  (44.60-66.25) | 25.00  (16.94-35.27) | 82.50  (76.26-87.37) |
| Senior radiologist2 | 0.555  (45.34-65.74) | 27.27  (16.60-41.41) | 82.11  (77.27-86.09) |
| Senior pulmonologist | 0.591  (50.57-67.61) | 54.55  (28.42-78.39) | 82.91  (79.64-85.74) |
| Joint AI model | 0.740  (63.56-84.41) | 31.25  (14.76-54.41) | 81.25  (78.02-84.10) |

**Supplementary Table. 2** | Comparison of sensitivity between Joint AI model and human readers. Data in parenthesis are 95% CI. n=34 for UIP analysis; n=10 for CHP analysis; n=44 for NSIP analysis; n=14 for sarcoidosis analysis; n= 10 for other ILD analysis. Two-sided P values were caculated.

1. UIP

| Senior Thoracic radiologist | 55.88  (37.89, 72.81) | p<0.05 |
| --- | --- | --- |
| Junior thoracic radiologist1 | 41.18  (24.65, 59.30) | p<0.001 |
| Junior thoracic radiologist2 | 47.06  (29.8, 64.87) | p<0.001 |
| Thoracic radiology fellow | 70.59  (52.52, 84.90) | p=0.34 |
| Senior general radiologist1 | 52.94  (35.13, 70.22) | p<0.01 |
| Senior general radiologist2 | 29.41  (15.10, 47.48) | p<0.001 |
| Senior pulmonologist | 41.18  (24.65, 59.30) | p<0.001 |
| Joint AI model | 82.35  (65.47, 93.24) |  |

1. CHP

| Senior Thoracic radiologist | 50.00  (18.71, 81.29) | p=0.38 |
| --- | --- | --- |
| Junior thoracic radiologist1 | 30.00  (6.67, 62.25) | p=1 |
| Junior thoracic radiologist2 | 20.00  (2.52, 55.61) | p=1 |
| Thoracic radiology fellow | 40.00  (12.16, 73.76) | p=0.63 |
| Senior general radiologist1 | 10.00  (0.25, 44.50) | p=1 |
| Senior general radiologist2 | 10.00  (0.25, 44.50) | p=1 |
| Senior pulmonologist | 40.00  (12.16, 73.76) | p=0.63 |
| Joint AI model | 20.00  (2.52, 55.64) |  |

1. NSIP

| Senior Thoracic radiologist | 54.55  (38.85, 69.61) | p=0.33 |
| --- | --- | --- |
| Junior thoracic radiologist1 | 50.00  (34.56, 65.44) | p=0.14 |
| Junior thoracic radiologist2 | 47.73  (32.46, 63.31) | p=0.10 |
| Thoracic radiology fellow | 52.27  (36.69, 67.54) | p=0.24 |
| Senior general radiologist1 | 29.55  (16.73, 45.20) | p<0.01 |
| Senior general radiologist2 | 65.91  (50.08, 79.51) | p=1 |
| Senior pulmonologist | 79.55  (64.70, 90.20) | p=0.11 |
| Joint AI model | 65.91  (50.08, 79.51) |  |

1. Sarcoidosis

| Senior Thoracic radiologist | 35.71  (12.76, 64.86) | p=1 |
| --- | --- | --- |
| Junior thoracic radiologist1 | 42.86  (17.66, 71.14) | p=1 |
| Junior thoracic radiologist2 | 42.86  (17.66, 71.14) | p=1 |
| Thoracic radiology fellow | 42.86  (17.66, 71.14) | p=1 |
| Senior general radiologist1 | 42.86  (17.66, 71.14) | p=1 |
| Senior general radiologist2 | 35.71  (12.76, 64.86) | p=1 |
| Senior pulmonologist | 35.71  (12.76, 64.86) | p=1 |
| Joint AI model | 42.86  (17.66, 71.14) |  |

1. Other ILD

| Senior Thoracic radiologist | 53.85  (33.37, 73.41) | p<0.05 |
| --- | --- | --- |
| Junior thoracic radiologist1 | 38.46  (20.23, 59.43) | p=0.18 |
| Junior thoracic radiologist2 | 42.31  (23.35, 63.08) | p=0.11 |
| Thoracic radiology fellow | 42.31  (23.35, 63.08) | p=0.15 |
| Senior general radiologist1 | 46.15  (26.59, 66.63) | p=0.09 |
| Senior general radiologist2 | 34.62  (17.21, 55.67) | p=0.39 |
| Senior pulmonologist | 23.08  (8.97, 43.65) | p=1 |
| Joint AI model | 19.23  (6.55, 39.35) |  |

**Supplementary Table. 3** | Comparison of specificity between Joint AI model and human readers. Data in parenthesis are 95% CI. n=34 for UIP analysis; n=10 for CHP analysis; n=44 for NSIP analysis; n=14 for sarcoidosis analysis; n= 10 for other ILD analysis. Two-sided P values were caculated.

1. UIP

| Senior Thoracic radiologist | 96.81  (90.96, 99.34) | p<0.001 |
| --- | --- | --- |
| Junior thoracic radiologist1 | 95.74  (89.46, 98.83) | p<0.001 |
| Junior thoracic radiologist2 | 93.62  (86.62, 97.62) | p<0.001 |
| Thoracic radiology fellow | 91.49  (83.92, 96.25) | p<0.001 |
| Senior general radiologist1 | 81.91  (72.63, 89.10) | p<0.05 |
| Senior general radiologist2 | 95.74  (89.46, 98.83) | p<0.001 |
| Senior pulmonologist | 94.68  (88.02, 98.25) | p<0.001 |
| Joint AI model | 68.09  (57.67, 77.33) |  |

1. CHP

| Senior Thoracic radiologist | 86.44  (78.92, 92.05) | p<0.01 |
| --- | --- | --- |
| Junior thoracic radiologist1 | 83.90  (76.00, 90.02) | p<0.001 |
| Junior thoracic radiologist2 | 84.75  (76.97, 90.70) | p<0.001 |
| Thoracic radiology fellow | 89.83  (82.91, 94.63) | p<0.05 |
| Senior general radiologist1 | 92.37  (86.01, 96.45) | p=0.15 |
| Senior general radiologist2 | 86.44  (78.92, 92.05) | p<0.01 |
| Senior pulmonologist | 84.75  (76.97, 90.70) | p<0.001 |
| Joint AI model | 97.46  (92.75, 99.47) |  |

1. NSIP

| Senior Thoracic radiologist | 85.71  (76.38, 92.39) | p=0.48 |
| --- | --- | --- |
| Junior thoracic radiologist1 | 76.19  (65.65, 84.81) | p<0.05 |
| Junior thoracic radiologist2 | 76.19  (65.65, 84.81) | p<0.05 |
| Thoracic radiology fellow | 83.33  (73.62, 90.58) | p=0.24 |
| Senior general radiologist1 | 92.86  (85.10, 97.33) | p=0.79 |
| Senior general radiologist2 | 65.48  (54.31, 75.52) | p<0.001 |
| Senior pulmonologist | 57.14  (45.88, 67.89) | p<0.001 |
| Joint AI model | 90.48  (82.09, 95.80) |  |

1. Sarcoidosis

| Senior Thoracic radiologist | 99.12  (95.21, 99.98) | p=0.13 |
| --- | --- | --- |
| Junior thoracic radiologist1 | 97.37  (92.50, 99.45) | p=0.45 |
| Junior thoracic radiologist2 | 98.25  (93.81, 99.79) | p=0.29 |
| Thoracic radiology fellow | 97.37  (92.50, 99.45) | p=0.51 |
| Senior general radiologist1 | 91.23  (84.46, 95.71) | p=0.42 |
| Senior general radiologist2 | 99.12  (95.21, 99.98) | p=0.13 |
| Senior pulmonologist | 100.00  (96.82, 100.00) | p<0.05 |
| Joint AI model | 94.74  (88.90, 98.04) |  |

1. Other ILD

| Senior Thoracic radiologist | 71.57  (61.78, 80.06) | p<0.001 |
| --- | --- | --- |
| Junior thoracic radiologist1 | 73.53  (63.87, 81.78) | p<0.01 |
| Junior thoracic radiologist2 | 74.51  (64.92, 82.62) | p<0.01 |
| Thoracic radiology fellow | 77.45  (68.11, 85.14) | p<0.05 |
| Senior general radiologist1 | 64.71  (54.62, 73.91) | p<0.001 |
| Senior general radiologist2 | 76.47  (67.04, 84.31) | p<0.05 |
| Senior pulmonologist | 95.10  (88.93, 98.39) | p=0.11 |
| Joint AI model | 89.22  (81.52, 94.49) |  |

**Supplementary Table. 4** | Detailed descriptions of the quantitative features and its correlation with 3-year survival rate. n=34 for UIP analysis; n=10 for CHP analysis; n=44 for NSIP analysis; n=14 for sarcoidosis analysis; n= 10 for other ILD analysis. Two-sided P values were caculated.

| features | p value | features | p value | features | p value | features | p value |
| --- | --- | --- | --- | --- | --- | --- | --- |
| Home_O2 | 0.182 | original_firstorder_Median | 0.579 | original_gldm_LargeDependenceLowGrayLevelEmphasis | 0.688 | original_ngtdm_Contrast | 0.825 |
| Occupation | 0.652 | original_firstorder_Minimum | 0.315 | original_gldm_LowGrayLevelEmphasis | 0.292 | original_ngtdm_Strength | 0.102 |
| CurrentSmoker | 0.276 | original_firstorder_RobustMeanAbsoluteDeviation | 0.816 | original_gldm_SmallDependenceEmphasis | 0.381 | age | 0.042 |
| FormerSmoker | 0.033 | original_firstorder_RootMeanSquared | 0.492 | original_gldm_SmallDependenceHighGrayLevelEmphasis | 0.750 | kvp | 0.165 |
| Hx_disease | 0.703 | original_firstorder_Skewness | 0.967 | original_gldm_SmallDependenceLowGrayLevelEmphasis | 0.253 | manufacturer | 0.614 |
| pulm_HTN | 0.785 | original_firstorder_Uniformity | 0.139 | original_glrlm_GrayLevelNonUniformity | 0.020 | thickness | 0.311 |
| Biopsy | 0.564 | original_firstorder_Variance | 0.014 | original_glrlm_GrayLevelNonUniformityNormalized | 0.008 | CNN_feature_0 | 0.768 |
| sex | 0.864 | original_glcm_Autocorrelation | 0.217 | original_glrlm_GrayLevelVariance | 0.475 | CNN_feature_1 | 0.847 |
| Med_label | 0.144 | original_glcm_ClusterProminence | 0.317 | original_glrlm_HighGrayLevelRunEmphasis | 0.524 | CNN_feature_2 | 0.024 |
| Therapeutic_label | 0.007 | original_glcm_ClusterShade | 0.098 | original_glrlm_LongRunEmphasis | 0.965 | CNN_feature_3 | 0.633 |
| FEV1 | 0.852 | original_glcm_ClusterTendency | 0.160 | original_glrlm_LongRunHighGrayLevelEmphasis | 0.953 | CNN_feature_4 | 0.052 |
| FVC | 0.899 | original_glcm_Contrast | 0.160 | original_glrlm_LongRunLowGrayLevelEmphasis | 0.947 | CNN_feature_5 | 0.099 |
| FEV1.FVC | 0.187 | original_glcm_Correlation | 0.829 | original_glrlm_LowGrayLevelRunEmphasis | 0.068 | CNN_feature_6 | 0.320 |
| DLCO | 0.972 | original_glcm_DifferenceAverage | 0.211 | original_glrlm_RunEntropy | 0.523 | CNN_feature_7 | 0.246 |
| timespan | 0.798 | original_glcm_DifferenceEntropy | 0.276 | original_glrlm_RunLengthNonUniformity | 0.147 | CNN_feature_8 | 0.484 |
| diagnostics_Image.original_Mean | 0.185 | original_glcm_DifferenceVariance | 0.189 | original_glrlm_RunLengthNonUniformityNormalized | 0.980 | CNN_feature_9 | 0.231 |
| diagnostics_Image.original_Maximum | 0.743 | original_glcm_Id | 0.234 | original_glrlm_RunPercentage | 0.033 | CNN_feature_10 | 0.177 |
| diagnostics_Mask.original_VoxelNum | 0.729 | original_glcm_Idm | 0.237 | original_glrlm_RunVariance | 0.950 | CNN_feature_11 | 0.075 |
| diagnostics_Mask.original_VolumeNum | 0.471 | original_glcm_Idmn | 0.419 | original_glrlm_ShortRunEmphasis | 0.117 | CNN_feature_12 | 0.543 |
| original_shape_Elongation | 0.761 | original_glcm_Idn | 0.590 | original_glrlm_ShortRunHighGrayLevelEmphasis | 0.422 | CNN_feature_13 | 0.873 |
| original_shape_Flatness | 0.566 | original_glcm_Imc1 | 0.743 | original_glrlm_ShortRunLowGrayLevelEmphasis | 0.180 | CNN_feature_14 | 0.320 |
| original_shape_LeastAxisLength | 0.748 | original_glcm_Imc2 | 0.042 | original_glszm_GrayLevelNonUniformity | 0.035 | CNN_feature_15 | 0.061 |
| original_shape_MajorAxisLength | 0.706 | original_glcm_InverseVariance | 0.233 | original_glszm_GrayLevelNonUniformityNormalized | 0.707 | CNN_feature_16 | 0.572 |
| original_shape_Maximum2DDiameterColumn | 0.725 | original_glcm_JointAverage | 0.177 | original_glszm_GrayLevelVariance | 0.156 | CNN_feature_17 | 0.603 |
| original_shape_Maximum2DDiameterRow | 0.725 | original_glcm_JointEnergy | 0.393 | original_glszm_HighGrayLevelZoneEmphasis | 0.744 | CNN_feature_18 | 1.000 |
| original_shape_Maximum2DDiameterSlice | 0.917 | original_glcm_JointEntropy | 0.687 | original_glszm_LargeAreaEmphasis | 0.675 | CNN_feature_19 | 0.029 |
| original_shape_Maximum3DDiameter | 0.743 | original_glcm_MCC | 0.679 | original_glszm_LargeAreaHighGrayLevelEmphasis | 0.415 | CNN_feature_20 | 0.148 |
| original_shape_MeshVolume | 0.748 | original_glcm_MaximumProbability | 0.293 | original_glszm_LargeAreaLowGrayLevelEmphasis | 0.402 | CNN_feature_21 | 0.612 |
| original_shape_MinorAxisLength | 0.792 | original_glcm_SumAverage | 0.177 | original_glszm_LowGrayLevelZoneEmphasis | 0.428 | CNN_feature_22 | 0.249 |
| original_shape_Sphericity | 0.558 | original_glcm_SumEntropy | 0.060 | original_glszm_SizeZoneNonUniformity | 0.050 | CNN_feature_23 | 0.226 |
| original_shape_SurfaceArea | 0.980 | original_glcm_SumSquares | 0.160 | original_glszm_SizeZoneNonUniformityNormalized | 0.547 | CNN_feature_24 | 0.544 |
| original_shape_SurfaceVolumeRatio | 0.088 | original_gldm_DependenceEntropy | 0.909 | original_glszm_SmallAreaEmphasis | 0.565 | CNN_feature_25 | 0.164 |
| original_firstorder_10Percentile | 0.292 | original_gldm_DependenceNonUniformity | 0.648 | original_glszm_SmallAreaHighGrayLevelEmphasis | 0.788 | CNN_feature_26 | 0.914 |
| original_firstorder_90Percentile | 0.240 | original_gldm_DependenceNonUniformityNormalized | 0.199 | original_glszm_SmallAreaLowGrayLevelEmphasis | 0.547 | CNN_feature_27 | 0.297 |
| original_firstorder_Energy | 0.075 | original_gldm_DependenceVariance | 0.756 | original_glszm_ZoneEntropy | 0.624 | CNN_feature_28 | 0.734 |
| original_firstorder_Entropy | 0.282 | original_gldm_GrayLevelNonUniformity | 0.239 | original_glszm_ZonePercentage | 0.933 | CNN_feature_29 | 0.322 |
| original_firstorder_InterquartileRange | 0.018 | original_gldm_GrayLevelVariance | 0.020 | original_glszm_ZoneVariance | 0.681 | CNN_feature_30 | 0.291 |
| original_firstorder_Kurtosis | 0.865 | original_gldm_HighGrayLevelEmphasis | 0.223 | original_ngtdm_Busyness | 0.354 | CNN_feature_31 | 0.210 |
| original_firstorder_MeanAbsoluteDeviation | 0.633 | original_gldm_LargeDependenceEmphasis | 0.058 | original_ngtdm_Coarseness | 0.165 | time_filter | 0.580 |
| original_firstorder_Mean | 0.531 | original_gldm_LargeDependenceHighGrayLevelEmphasis | 0.892 | original_ngtdm_Complexity | 0.943 | average_timespan | 0.034 |
| diagnostics_Image_original_Minimum | N/A | original_shape_VoxelVolume | N/A | original_firstorder_Range | N/A | original_firstorder_Maximum | N/A |
| original_firstorder_TotalEnergy | N/A |  |  |  |  |  |  |

**Supplementary Table 5 | Details of medications and therapeutic classes within each ILD subtype**

| Medications (Therapeutic Class) | UIP (n=132) | CHP (n=37) | NSIP (n=142) | SAR (n=42) | Other (n=96) |
| --- | --- | --- | --- | --- | --- |
| Azathioprine (immunosuppressant) |  |  |  |  |  |
| Yes | 1 (0.1%) | 1 (2.7%) | 5 (3.5%) | 0 (0%) | 0 (0%) |
| No | 131 (99.2%) | 36 (97.3%) | 137 (96.5%) | 42 (100%) | 96 (100%) |
| Bosentan (Cardiovascular) |  |  |  |  |  |
| Yes | 0 (0%) | 0 (0%) | 1 (0.7%) | 0 (0%) | 0 (0%) |
| No | 132 (100%) | 37 (100%) | 141 (99.3%) | 42 (100%) | 96 (100%) |
| Cyclophosphamide (Antineoplastics) |  |  |  |  |  |
| Yes | 0 (0%) | 0 (0%) | 1 (0.7%) | 0 (0%) | 0 (0%) |
| No | 132 (100%) | 37 (100%) | 141 (99.3%) | 42 (100%) | 96 (100%) |
| Mycophenolate (immunosuppressant) |  |  |  |  |  |
| Yes | 2 (1.5%) | 4 (10.8%) | 25 (17.6%) | 0 (0%) | 8 (8.3%) |
| No | 130 (98.5%) | 33 (89.2%) | 117 (82.4%) | 42 (100%) | 88 (91.7%) |
| Nintedanib (Other) |  |  |  |  |  |
| Yes | 10 (7.6%) | 0 (0%) | 0 (0%) | 0 (0%) | 0 (0%) |
| No | 122 (92.4%) | 37 (100%) | 142 (100%) | 42 (100%) | 96 (100%) |
| Pirfenidone (Other) |  |  |  |  |  |
| Yes | 15 (11.4%) | 1 (2.7%) | 0 (0%) | 0 (0%) | 1 (1.0%) |
| No | 117 (88.6%) | 36 (97.3%) | 142 (100%) | 42 (100%) | 95 (99.0%) |
| Prednisone (Hormone) |  |  |  |  |  |
| Yes | 17 (12.9%) | 10 (27.0%) | 25 (17.6%) | 8 (19.0%) | 16 (16.7%) |
| No | 115 (87.1%) | 27 (73.0%) | 117 (82.4%) | 34 (81.0%) | 80 (83.3%) |
| Rituximab (Other) |  |  |  |  |  |
| Yes | 0 (0%) | 0 (0%) | 1 (0.7%) | 0 (0%) | 0 (0%) |
| No | 132 (100%) | 37 (100%) | 141 (99.3%) | 42 (100%) | 96 (100%) |
| Other (Other) |  |  |  |  |  |
| Yes | 87 (65.9%) | 21 (56.8%) | 84 (59.2%) | 34 (81.0%) | 71 (74.0%) |
| No | 45 (34.1%) | 16 (43.2%) | 58 (40.8%) | 8 (19.0%) | 25 (26.0%) |
