# Supplementary material for: Interstitial lung disease diagnosis and prognosis using an AI system integrating longitudinal data
Source: Nat Commun. 2023 Apr 20;14:2272. doi: 10.1038/s41467-023-37720-5 (PMC10119160; doi:10.1038/s41467-023-37720-5)
Supplement: Supplementary file 3 — Reporting Summary [file 41467_2023_37720_MOESM3_ESM.pdf]

nature portfolio

Corresponding author(s): Yang Yang, Ph.D.

Last updated by author(s): 02/28/2023

Reporting Summary

Nature Portfolio wishes to improve the reproducibility of the work that we publish. This form provides structure for consistency and transparency in reporting. For further information on Nature Portfolio policies, see our Editorial Policies and the Editorial Policy Checklist.

Statistics

For all statistical analyses, confirm that the following items are present in the figure legend, table legend, main text, or Methods section.

n/a	Confirmed	
☐	☒	The exact sample size (n) for each experimental group/condition, given as a discrete number and unit of measurement
☐	☒	A statement on whether measurements were taken from distinct samples or whether the same sample was measured repeatedly
☐	☒	The statistical test(s) used AND whether they are one- or two-sided<br>*Only common tests should be described solely by name; describe more complex techniques in the Methods section.*
☒	☐	A description of all covariates tested
☒	☐	A description of any assumptions or corrections, such as tests of normality and adjustment for multiple comparisons
☐	☒	A full description of the statistical parameters including central tendency (e.g. means) or other basic estimates (e.g. regression coefficient) AND variation (e.g. standard deviation) or associated estimates of uncertainty (e.g. confidence intervals)
☐	☒	For null hypothesis testing, the test statistic (e.g. F, t, r) with confidence intervals, effect sizes, degrees of freedom and P value noted<br>*Give P values as exact values whenever suitable.*
☒	☐	For Bayesian analysis, information on the choice of priors and Markov chain Monte Carlo settings
☒	☐	For hierarchical and complex designs, identification of the appropriate level for tests and full reporting of outcomes
☐	☒	Estimates of effect sizes (e.g. Cohen's d, Pearson's r), indicating how they were calculated

Our web collection on statistics for biologists contains articles on many of the points above.

Software and code

Policy information about availability of computer code

Data collection	GE Centricity Universal Viewer and Viewer 6.0: DICOM viewer for reading CT data. Clinical variables were collected on EPIC.
Data analysis	Pandas (version 1.0.1): open source library Pandas was used for tabular data<br>Matplotlib (version 3.1.3): open source library Matplotlib was used for making plots<br>sklearn (version 0.24.1): open source library scikit-learn was used for metrics such as AUC and machine learning classifier training<br>Tensorflow (version 2.4.0): open source library Tensorflow was used to train deep learning models<br>Numpy (version 1.19.5): open source library Numpy was used in imaging preprocessing<br>Statsmodels (version 0.13.2): a python library Statsmodels was used to calculate the confidence intervals and p-values for sensitivity, specificity and accuracy<br>pROC (version 1.18.0): library pROC in R was used to calculate the confidence intervals for AUC and calculate the p-values of AUC<br>DTComPair (version 1.0.3): a library in R was used to calculate the two-sided p-values of predictive values<br>Python (version 3.8.10)<br>R (version 4.1.3)<br>All the codes we used to train the models have been posted in this github repository https://github.com/lzl199704/ILD.

For manuscripts utilizing custom algorithms or software that are central to the research but not yet described in published literature, software must be made available to editors and reviewers. We strongly encourage code deposition in a community repository (e.g. GitHub). See the Nature Portfolio guidelines for submitting code & software for further information.

Data

Policy information about availability of data

All manuscripts must include a data availability statement. This statement should provide the following information, where applicable:

- Accession codes, unique identifiers, or web links for publicly available datasets
- A description of any restrictions on data availability
- For clinical datasets or third party data, please ensure that the statement adheres to our policy

The in-house datasets generated and/or analysed during the current study are not publicly available due to HIPAA compliance and were used with Mount Sinai institutional permission for the purposes of this project. All requests for access to in-house data will be addressed to the corresponding authors, Dr. Xueyan Mei (xueyan.mei@icahn.mssm.edu), Dr. Yang Yang (yang.yang4@ucsf.edu) or Dr. Zahi Fayad (zahi.fayad@mssm.edu), and will be processed in accordance with Mount Sinai institutional guidelines. Mount Sinai Innovation Partners (MSIP) will assess all requests based on the purpose of data request, and it may take up to one month to process the request.
A material-transfer or data-usage agreement will be required between Mount Sinai and the receiving organization. The requesting organization must provide comprehensive details, including the name and full contact information of the individual and institution making the request, along with specific identification of the data being requested. Additionally, the requesting organization must clearly state the intended purpose of the data transfer and provide assurances that the transferred data will only be used for non-commercial academic and educational purposes in compliance with Mount Sinai institutional guidelines.
The pretrained models used in this paper are available at https://doi.org/10.1148/ryai.210315.
Source data are provided as a Source Data file.

Human research participants

Policy information about studies involving human research participants and Sex and Gender in Research.

Reporting on sex and gender	Patient's sex was collected in this study. Sex information was used in the diagnosis of ILD subtypes as well as the prediction of three year survival analysis. A total of 226 males and 223 females diagnosed with ILD were inlucded in this study.
Population characteristics	A total of 449 patients who diagnosed with interstitial lung disease enrolled in the registry. The patient population age ranged from 22 to 91 years (median 63, IQR 56-71), with 226 males and 223 females. A total of 132 patients (29.4%) were diagnosed with usual interstitial pneumonia (UIP), 37 patients (8.2%) with chronic hypersensitivity pneumonitis (CHP), 142 patients (31.6%) with nonspecific interstitial pneumonia (NSIP), 42 patients (9.4%) with sarcoidosis and 96 patients (21.4%) with other various ILD.
Recruitment	We collected CT scans and associated clinical information from 449 patients enrolled from 2014 to April 2021. Individuals for participation in Mount Sinai Medical Center Research Registry for Interstitial Lung Disease (MSMC-ILD) included all adult (age > 18 years old) patients who were receiving or seeking medical care for the treatment of interstitial lung disease at Mount Sinai Medical Center, St Luke's and Beth Israel Medical Centers. Patients with lung fibrosis or other interstitial lung disease were enrolled in the MSMC-ILD and assessing the extent of the disease. MSMC-ILD was established in 2014. The diagnosis of an ILD subtype followed the ATS2018 guidelines. All registry patients had a consensus diagnosis from radiology, pathology, and pulmonology. In this study, occupational exposure or other environmental exposure is included as a clinical feature. It is likely that the patient cohort at MSMC might be different from other patient cohorts. For example, patients at MSMC might be influenced by World Trade Center exposure. Study participants did not receive compensation
Ethics oversight	The study was approved by the Institutional Review Board (IRB) of the Mount Sinai School of Medicine, in accordance with Mount Sinai's Federal Wide Assurances to the Department of Health and Human Services (ID# STUDY-14-00584-CR001). Written informed consent has been obtained from patients enrolled in this research registry. A Data and Safety Monitoring Board (DSMB) from Mount Sinai IRB had oversight of the study.

Note that full information on the approval of the study protocol must also be provided in the manuscript.

Field-specific reporting

Please select the one below that is the best fit for your research. If you are not sure, read the appropriate sections before making your selection.

☒ Life sciences ☐ Behavioural & social sciences ☐ Ecological, evolutionary & environmental sciences

For a reference copy of the document with all sections, see nature.com/documents/nr-reporting-summary-flat.pdf

Life sciences study design

All studies must disclose on these points even when the disclosure is negative.

Sample size	The MSMC-ILD registry was a multi-center cohort. Of the 449 patients in the MSMC-ILD, 128 who had their initial scan and pulmonary function test performed at the Mount Sinai Hospital (MSH) were used as the holdout testing set. The remaining 321 patients who underwent CT in non-MSH centers were randomly split into a training set (80.4%, 258 cases with 78 UIP) and a validation set (19.6%, 63 cases with 20 UIP). All the cases in this cohort were independent and non-repeating samples. The MSH test set was hold-out for the comparison between human readers and AI models. We believe the sample size was sufficient for the test set because it can be considered as external evaluation of the models where the CT images and clinical information were collected from MSH while the training and validation datasets were

collected from non-MSH centers.

Data exclusions	The data exclusion criteria was pre-established. Men and women greater than 18 years of age with diagnosis of ILD were included in the registry. Those not meeting the inclusion criteria were excluded. For the diagnosis of ILD subtypes via the initial scan, we excluded data when CT images have low equality. A total of 9 CT scans was excluded because of low image quality. For the prediction of three-year survival by using all available longitudinal data, 224 patients who had incomplete follow up information within three years were excluded.
Replication	We created a hold-out external test set to evaluate the performance of the deep learning model. The test set of 128 cases was unseen by the models. The comparison between human readers and the best AI model to diagnose ILD subtypes were performed on this unseen test set that all cases were independent and non-repeated samples. For the comparison between long short-term memory (LSTM) and Transformer models in prediction of 3-year survival rate, we ran the models 30 times on the same datasets and compared the average are under the receiver operating characteristics curve between LSTM and Transformer models.
Randomization	Patients who underwent CT in non-MSH centers were randomly split into a training set (80.4%, 258 cases with 78 UIP) and a validation set (19.6%, 63 cases with 20 UIP). Patient who underwent CT at MSH were used as external evaluation test set (128 cases with 34 UIP).
Blinding	For the comparisons between human readers and AI system in diagnosing ILD subtypes, all readers were provided with the same deidentified lung CT scans and clinical information. The readers were blinded to the diagnosis of the patients to have a fair comparison to AI models.

Reporting for specific materials, systems and methods

We require information from authors about some types of materials, experimental systems and methods used in many studies. Here, indicate whether each material, system or method listed is relevant to your study. If you are not sure if a list item applies to your research, read the appropriate section before selecting a response.

Materials & experimental systems

n/a	Involved in the study	
☒	☐	Antibodies
☒	☐	Eukaryotic cell lines
☒	☐	Palaeontology and archaeology
☒	☐	Animals and other organisms
☒	☐	Clinical data
☒	☐	Dual use research of concern

Methods

n/a	Involved in the study	
☒	☐	ChIP-seq
☒	☐	Flow cytometry
☒	☐	MRI-based neuroimaging
